# Corrugations in Free-Standing Graphene

**DOI:** 10.3390/nano12203562

**Published:** 2022-10-11

**Authors:** Rajendra Singh, Daniel Scheinecker, Ursula Ludacka, Jani Kotakoski

**Affiliations:** Faculty of Physics, University of Vienna, Boltzanngasse 5, 1090 Vienna, Austria

**Keywords:** graphene, electron microscopy, corrugations, electron diffraction

## Abstract

Although both the tendency of 2D materials to bend out of plane as well as its effect on materials’ properties are well known, the factors influencing this phenomenon have not been extensively studied. Graphene, the one-atom-thick membrane of carbon atoms, is both arguably the best known 2D material, as well as the most prone to spontaneous corrugations. Here, we use electron diffraction to systematically study the factors influencing corrugations in graphene, including the size of the free-standing area, the preparation method, the amount of surface contamination, and electron-beam-induced structural disorder. We find that mechanically exfoliated graphene is less corrugated than graphene grown via chemical vapor deposition (corrugation amplitude of (0.83±0.10) Å compared to (1.33±0.20) Å for a free-standing area with a diameter of 1.7μm). Similarly, corrugation amplitude grows by more than a factor of two when the diameter of the free- standing area is increased from 1.7μm to ca. 3.0μm. Electron beam irradiation affects the corrugation in two ways, firstly by removing the hydrocarbon contamination, which decreases corrugation, and secondly by creating increasing amounts of disorder into the material, which again increases corrugation. Overall, our results show that control over the sample during both initial preparation and post-preparation treatment allows for a change in the amount of corrugation in free-standing 2D materials, which may lead to new advances in their use in applications.

## 1. Introduction

Since the isolation of graphene in 2004, it and other 2D materials have been considered for a number of application fields ranging from transistors [1], sensors [2,3], and photo-detectors, [4] to battery electrodes [5] and valleytronics [6]. Unlike conventional crystalline materials, where atomic planes in a defect-free bulk are flat and deviations from this are expected only at the surface, 2D materials are known to assume a non-flat structure. This can either result from roughness of the underlying surface, or arise spontaneously in free-standing samples due to sample preparation or out-of-plane phonon motion. The resulting corrugations have an influence on the properties of the individual 2D materials [7,8,9,10,11,12], and may also govern the properties of the inter-material interfaces in van der Waals heterostructures [13] that are becoming increasingly important for applications [14]. Therefore, means to exploit the out-of-plane structure of 2D materials have been sought for, as described in detail in Ref. [15]. As an example, corrugations introduced via defects [16] or intercalation [17] have been shown to lead to a Kekulé distortion (a bond density wave instability) that breaks the lattice symmetry, with interesting implications for the electronic properties of graphene.

However, despite their importance, intrinsic corrugations of 2D materials have not yet been extensively studied, due to the experimental challenges involved—the method for measuring corrugations must have a sub-Ångström accuracy, while at the same time interacting weakly enough with the 2D material to not influence its structure. This rules out, for example, scanning probe methods, since the interaction with the probe is known to influence the shape of the 2D material [18,19]. In contrast, as was shown by Meyer et al. in 2007, transmission electron microscopy (TEM) diffraction measurements fulfil these conditions for free-standing samples [20]. Nevertheless, only a handful of studies have reported measurements on the intrinsic corrugations in 2D materials using this method [21,22,23], and therefore, it remains an open question as to how they are influenced, for example, by sample type, the area of the suspended sample, and other factors.

Here, we prepare free-standing single-layer graphene, both via mechanical exfoliation and from samples grown via chemical vapor deposition (CVD), and measure their out-of-plane corrugations based on the electron diffraction method. Crucially, we establish the influence of the sample type and the size of the suspended area on the results. We show that mechanically exfoliated samples have a corrugation amplitude that is ca. 36% smaller than that of CVD samples with the same size of the suspended area ((0.83±0.10) Å compared to (1.33±0.20) Å for a suspended area with a diameter of 1.7 μm), and increasing the diameter of the suspended area from 1.7 to 3.0 μm increases the amplitude for CVD samples to (2.10±0.27) Å. Finally, we also point out that the amount of ubiquitous hydrocarbon-based contamination on the suspended sample also plays a role. Using an electron beam to induce chemical etching, the amount of contamination on the sample is reduced, which leads to a significant decrease in the corrugation amplitude (by up to 21%) for increasing electron dose. However, this effect is counterbalanced by an increase in the corrugation amplitude that results from an increasing amount of disorder in the sample that begins to dominate when the etching starts to create pores in graphene, ultimately leading to more significant corrugations than those measured for the pristine sample. Overall, our results clearly demonstrate that corrugations in 2D materials can be influenced by the sample type, size of the suspended area, sample quality, and external factors such as surface contamination, opening the way for optimizing sample preparation to lead to the highest quality material for applications of both individual 2D materials, as well as their heterostructures.

## 2. Materials and Methods

Mechanically exfoliated samples were prepared from bulk highly oriented pyrolytic graphite (HOPG) using an adhesive tape, and transferred to an oxidised silicon (Si/SiO${}_{2}$) substrate. Si/SiO${}_{2}$ provides sufficient contrast to distinguish between single and multi-layer graphene flakes under a light microscope. After selecting the single-layer graphene flake, an Au 300 mesh Quantifoil TEM grid with 1.7 μm holes in a 10–12 nm thick amorphous carbon film was placed on the flake, with carbon film facing the graphene flake. Using a micro-manipulator, the TEM grid was pinned down to hold it at the desired place and to increase the contact surface area between the carbon film and the graphene flake. A drop of Iso-Propyl Alcohol (IPA) was dropped onto the TEM grid while holding the grid with the manipulator. IPA works as a mediator to bind the TEM grid with the underlying graphene flake during its evaporation process. Once the IPA is fully dried, the TEM grid with the flake was removed from the silicon substrate by partially etching the SiO${}_{2}$ layer with a KOH solution. Subsequently, samples were cleaned in de-ionized water and IPA to remove any remaining KOH and Si residuals.

Commercial samples were grown via chemical vapor deposition (CVD) on copper substrate, and transferred onto a 100 μm thick polymer film (EasyTransfer Graphene) by Graphenea Inc. TEM sample preparation was carried out in three steps following the user instructions provided by the company.

For comparing mechanically exfoliated samples to the CVD ones, sample carriers with hole sizes of ca. 1.7 μm were used, whereas for comparing the influence of the size of the freestanding area, we used sample carries with hole sizes varying from 1.7 μm to 3.0 μm. Note that all holes are in reality slightly elliptical, and the number used here corresponds to the average of the shorter and the longer diameters of the holes. All samples and the main results are listed in Table 1.

**Figure 1:**
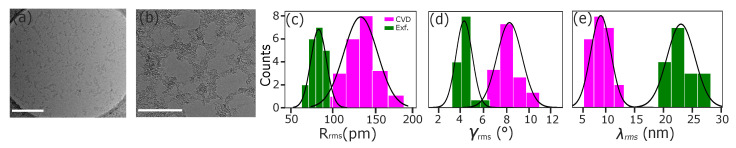
**Corrugations in exfoliated and CVD-grown graphene.** (**a**,**b**) Overview and high magnification TEM images of a CVD graphene sample. Scale bars are 300 nm and 30 nm, respectively. (**c**–**e**) Roughness/corrugation parameters; root-mean-square of corrugation height $\left(R_{rms}\right)$, normal inclination $\left(\gamma_{rms}\right)$, and the corrugation length $\left(\lambda_{rms}\right)$ for the exfoliated (green) and CVD (magenta) graphene samples. Black lines show the normal distributions corresponding to the mean and standard deviation of the data. The suspended area was 1.7 μm in diameter for both CVD and exfoliated samples.

The electron diffraction patterns were recorded with an FEI Titan 80–300 microscope at an acceleration voltage of 80 kV. For each measured area, the area was first identified to have a continuous graphene sheet covering the complete opening in the sample carrier, and not having any obvious irregularities, such as grain boundaries, folds, or excessive contamination. Examples of bright-field phase contrast images of measured areas are shown in Figure 1. Next, diffraction patterns were recorded, while taking care to minimize the electron dose on the sample during all steps of the process. The estimated beam current was ca. 40–42 $e^{-}/$nm${}^{2}/$s, resulting in an estimated dose of $4\times 10^{3}\;e^{-}/$nm${}^{2}$ per diffraction pattern. The aberration parameters (coma (B1): 35−40 nm, two-fold astigmatism (A2): 40–50 nm, two-fold spherical aberration (C3): 0–2 μm, three-fold astigmatism (A3), star-shape astigmatism (S3): ∼1 nm) were kept constant to minimize their influence in the measurements. Diffraction patterns were recorded from 0 to 30${}^{\circ }$ with a step size of 2${}^{\circ }$. After every tilt, the compu-stage was allowed to stabilize for ca. 8–10 s to avoid artificial broadening of the diffraction spots.

Measuring sample corrugation from diffraction patterns is based on the spreading of the diffraction spots. For a perfectly flat graphene, sharp peaks are formed due to the infinitely long and narrow relrods. For a corrugated sample, relrods corresponding to many infinitesimally small sample areas with different tilts due to local curvature sum up to a cone. Because the directions of the relrods depend on the orientation of each small area, the amount of opening of the cone depends on the amount of corrugation. For a non-tilted sample, the cones are intersected by the Ewald’s sphere (with a radius of ca. 250 nm${}^{-1}$ for 80-kV electrons, it can be assumed a plane for a diffraction pattern with a size of some 1/nm) at their narrowest point, whereas for a tilted sample, the cones are cut at heights depending on the tilt, and the corresponding diffraction spots appear at tilt-determined positions. Thereby recording diffraction patterns at different tilts allows for the reconstruction of a 3D map of the reciprocal space, which is further used to reconstruct the 3D shape of the sample.

After the diffraction patterns have been obtained, the root-mean-square amplitude of the corrugations $R_{rms}$, root-mean-square inclination $\gamma_{rms}$, and the corrugation wavelength λ can be calculated from the relationship of the maximum intensity of the diffraction spot *I* and its location in the reciprocal space *G*, as was shown by Thomsen et al. [22] as

(1) $$R_{rms}=\sqrt{{\langle h\rangle }^{2}}=\frac{1}{2\pi }\sqrt{\frac{-d\ln(I)}{dG^{2}}},\;\;\gamma_{rms}={\langle |\nabla h|\rangle }^{2},\;\;\mathrm{and}\;\;\frac{1}{\lambda }={\langle |\vec{q}|\rangle }^{2}=\frac{\sqrt{\gamma_{rms}}}{R_{rms}}.$$

For each sample type and size of the suspended area, a minimum of 20 measurements were carried out at independent sample locations to ensure sufficient statistics to allow for drawing conclusions from the results.

## 3. Results and Discussion

For the analysis of the tilt series, we mask each diffraction pattern so that only the first-order diffraction spots are visible, and we find the spot that shows the largest spread as a function of the tilt angle. The intensity *I* and the spot dispersion are measured by fitting the spot to the 2D Gaussian function, whereas the reciprocal lattice vector *G* is measured from the center of the diffraction patter. The center of the diffraction pattern is estimated by finding the coordinates of the opposite spots, and by taking the point exactly in the middle as the center. The value for $-d\ln\left(I\right)/dG^{2}$ is estimated through a linear fit.

We start by comparing the exfoliated and CVD-grown samples suspended over 1.7 μm holes, presented in Figure 1. As is clear from the mean values and standard deviations of the results, there is a remarkable difference between the two types of samples, with very little overlap in the distributions arising from the multiple measurements. Practically no measured area in the exfoliated sample has a corrugation amplitude as high as that of the least corrugated area of the CVD-grown sample, showing that the differing electronic [24,25] and mechanical [26] sample properties can not be reduced just to the existence of grain boundaries, but are likely also influenced by the overall 3D shape of the sample. For exfoliated graphene, we find $R_{rms}=\left(0.83\pm 0.10\right)$ Å, $\gamma_{rms}={\left(4.33\pm 0.71\right)}^{\circ }$ and λ=(23.81±2.47) nm, and for the CVD samples $R_{rms}=\left(1.33\pm 0.20\right)$ Å, $\gamma_{rms}={\left(8.23\pm 1.00\right)}^{\circ }$ and λ=(9.15±1.77) nm. All results are also listed in Table 1. Although this study does not reveal the reason for the higher corrugation amplitude in CVD-grown samples, this could be related to the surface roughness of the used Cu substrate as compared to an exfoliated flake that has grown inside a graphite crystal with an atomically flat interface between the neighboring layers.

**Figure 2:**
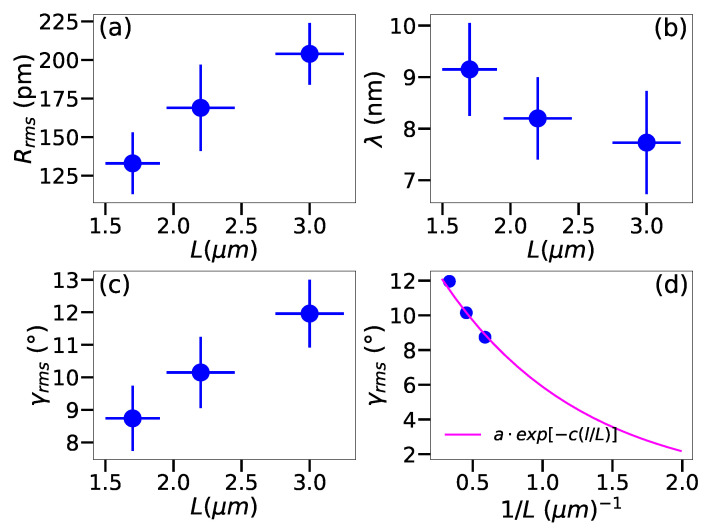
**Corrugations in CVD-grown graphene with different sizes of suspended area.** (**a**–**c**) Corrugation parameters for different sizes of the free-standing graphene area. The error bars correspond to the standard deviation of the data and the uncertainty in the size of the free-standing area arising from its ellipticity. (**d**) Root-mean-square inclination ($\gamma_{rms}$) as a function of the inverse size of the freestanding area, with a fit to a·exp[−c(l/L)], where *a* and *c* are fit parameters and *l* the electron coherence length.

Next, we move on to establish the relationship between the size of the free-standing area and the amount of corrugation in the sample. It has been suggested based on molecular dynamics simulations [27] that corrugations arising from the thermal motion of the lattice atoms leads to an exponential relationship between the fluctuations of the surface normal and the size of the suspended area *L*, which suggests $\gamma_{rms}\propto \exp\left(-l/L\right)$, where *l* is the size of the measured area that contributes to the diffraction pattern. The experimental results shown in Figure 2 and listed in Table 1 demonstrate a clear dependency of the corrugation on the size of the suspended area. Indeed, $R_{rms}$ increases from (1.33±0.20) Å for L=1.7 μm to (2.04±0.24) for L=3.0 μm, $\gamma_{rms}$ from ${\left(8.23\pm 1.00\right)}^{\circ }$ to ${\left(12.33\pm 2.32\right)}^{\circ }$, and λ decreases from (9.15±1.77) nm to (7.87±1.00) nm. Plotting $\gamma_{rms}$ as a function of *L* (Figure 2d) shows that the relationship is indeed exponential, following the prediction of Singh et al. [27], which suggests that the observed corrugations arise to a large extent from the thermal motion of the lattice atoms.

Finally, we also investigate the influence of the ubiquitous hydrocarbon-based surface contamination on the corrugations. Such contamination is present on all surfaces, but is typically of no relevance for the bulk properties of materials. However, due to all atoms being at the surface, 2D materials are susceptible to it, and because of the weak scattering potential of carbon nuclei, it is easily visible in TEM images of graphene. During TEM experiments, the energetic imaging electrons interact with any residual molecules in the microscope vacuum, which can lead to chemical changes in the sample, depending on the material and the composition of the residual vacuum. It has been previously shown [28,29] that at pressures typical to TEM instruments (ca. $10^{-7}$ mbar), such as the FEI Titan 80–300 used here, this leads to the chemical etching of carbon atoms that are not in an ideal bonding configuration. Since practically all clean areas of graphene are defect-free in typical samples, the first effect of the chemical etching is on the sample contamination, manifested by its disappearance.

**Figure 3:**
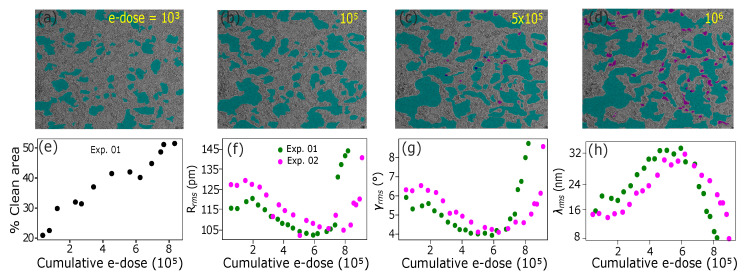
**Influence of contamination and disorder on the corrugations.** (**a**–**d**) TEM images of a graphene sample, recorded after a cumulative dose of $10^{3}$, $10^{5}$, $5\times 10^{5}$, and $10^{6}$$e^{-}/$nm${}^{2}$, respectively. The cyan area corresponds to clean graphene. As the cumulative electron dose increases from $10^{3}$ (image (**a**)) to $10^{5}$$e^{-}/$nm${}^{2}$ (image (**b**)), percentage of clean area also increases, whereas once the electron dose exceeds $5\times 10^{5}$$e^{-}/$nm${}^{2}$, chemical etching becomes more prominent, as seen in the increase in violet areas that correspond to pores. Field of view is ca. 100×100 nm${}^{2}$. (**e**) Clean area as a function of the cumulative electron dose. (**f**–**h**) Measured roughness parameters from two different sample areas as a function of the cumulative electron dose.

To study the influence of the contamination on corrugations, we carried out experiments where several subsequent tilt series were recorded, intercepted by exposing the suspended sample area to the electron beam for 5 min to gradually reduce the amount of contamination. We repeated the experiment at two different sample areas, leading in both cases to the same result (see Figure 3). Up to a cumulative electron dose of $10^{5}$ $e^{-}$/nm${}^{2}$, the contamination becomes thinner, but the clean sample area remains similar. In this regime, the etching has only a minor influence on the corrugations. However, after the clean area starts to increase when the etching continues, the sample starts to flatten significantly, which continues up to an electron dose of ca. $4-6\times 10^{5}$ $e^{-}$/nm${}^{2}$. At this point, the corrugation amplitude is ca. 79% from the value measured for the as-prepared sample. After this, the continuous etching reveals defects in the underlying graphene, which serve as seeding points for the etching process that starts to grow pores into the material itself. This disorder leads to increasing corrugation, countering the flattening caused by the removal of contamination, and finally results in a severely disordered and corrugated sample.

## 4. Conclusions

We demonstrated for the first time experimentally that both the sample type, as well as the size of the suspended area have a significant influence on the out-of-plane corrugations in single-layer graphene. Mechanically exfoliated graphene demonstrated a corrugation amplitude ((0.83±0.10) Å) that was 37% lower compared to the CVD-grown graphene ((1.33±0.20) Å), with the difference in corrugation wave length being even larger ((23.81±2.47) nm vs. (9.15±1.77) nm), for a suspended area with a diameter of L=1.7 μm. The size of the suspended area had a similar influence on corrugations, leading to an increase from (1.33±0.20) Å for L=1.7 μm to (2.04±0.24) Å for L=3.0 μm. The mean inclination was found to decay exponentially with l/L, indicating that the main cause for the measured corrugations is the thermal motion of the atoms. Although outside the scope of this study, it would be interesting to study the corrugation amplitude as a function of temperature to further explore the role of out-of-plane phonons in it. We also found that surface contamination leads to an increase in the corrugation amplitude, another matter that warrants future research. Nevertheless, the results provided here already highlight the need for efficient cleaning methods for device applications and for manufacturing van der Waals heterostructures of 2D materials. Finally, also disorder—in the form of nanopores created through chemical etching—were shown to lead to an increase in corrugations. Overall, our results provide the first experimental evidence of the different factors that influence corrugations in 2D materials that can serve as a basis for the fabrication of samples with improved performance for a number of different applications.

## Figures and Tables

**Table 1:** **Summary of results compared to literature values.**$R_{rms}$ is the root-mean-square amplitude of the corrugations, $\gamma_{rms}$ the root-mean-square inclination, and λ the corrugation wavelength. In total, six exfoliated samples were measured, whereas the CVD data is from two different samples. More than 20 measurements in independent sample areas were carried out for each parameter set.

Type	Size (μm)	$R_{\mathrm{rms}}$ (Å)	$\gamma_{\mathrm{rms}}$ (${}^{\circ }$)	λ (nm)
Exfoliated	1.7	0.83±0.10	4.34±0.71	23.81±2.47
CVD	1.7	1.33±0.20	8.23±1.00	9.15±1.77
CVD	2.2	1.69±0.30	9.81±2.09	8.20±2.60
CVD	3.0	2.04±0.24	12.33±2.32	7.87±1.00
Exfoliated [20]	1.0	-	5.0	25
Exfoliated [22]	-	1.14± 0.02	-	-
Exfoliated [23]	-	-	6.3	-
CVD [21]	-	1.7	6.0	10

## Data Availability

Data are available through an institutional repository at [30].
